# Cell membrane‐coated nanoparticles for the treatment of cancer

**DOI:** 10.1002/ctm2.1285

**Published:** 2023-05-30

**Authors:** Nishta Krishnan, Ronnie H. Fang, Liangfang Zhang

**Affiliations:** ^1^ Department of NanoEngineering Chemical Engineering Program, and Moores Cancer Center, University of California San Diego La Jolla California USA

**Keywords:** Cellular nanoparticle, Cell membrane coating, Drug delivery, Nanomedicine

1

Over the last two decades, nanomedicine has rapidly grown as a field that utilizes nanoscale materials for biomedical applications. With their ability to improve control over the localization and release of therapeutic agents, nanocarriers offer a safer and more effective approach for addressing a wide range of diseases.^1^ Along these lines, cell membrane coating is an emerging technology that has been leveraged to significantly improve the functionality of nanoparticulate platforms (Figure 1).^2^ Cell membrane‐coated nanoparticles (CNPs) possess many attributes that make them suitable for in vivo delivery applications, including improved biocompatibility, active targeting, increased stability, and reduced toxicity. As Fang et al. discussed recently in *Nature Reviews Clinical Oncology*, CNPs have become a promising tool for cancer treatment.^3^

**FIGURE 1:**
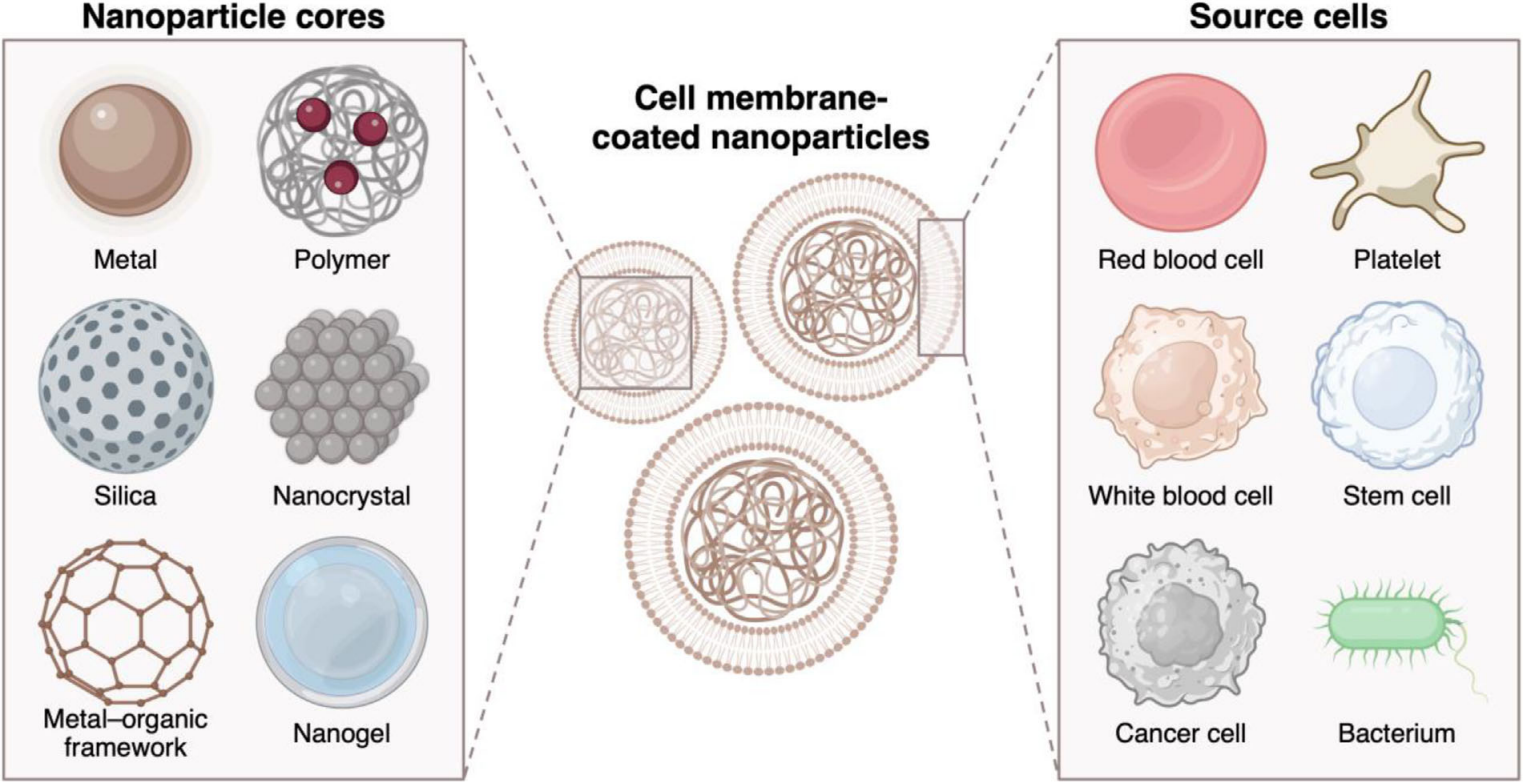
Cell membrane‐coated nanoparticles can be produced using different combinations of nanoparticle cores and cell membrane coatings. Each type of membrane coating offers unique properties and functionalities that are characteristic of the source cell. The material of the nanoparticle core can be chosen based on the desired application. Created with BioRender.

One major advantage of employing CNPs for drug delivery is their inherent biocompatibility. The naturally derived outer layer on these biomimetic nanoparticles greatly reduces the toxicity and immunogenicity concerns that oftentimes limit the clinical application of traditional synthetic platforms. The cell membrane coating serves as a camouflage that enables CNPs to be recognized as 'self', thus allowing them to evade detection and clearance by the immune system. This ultimately prolongs in vivo residence time and enables the nanoparticles to carry out their designated functions more effectively. The membrane can be derived from any cell type, including, but not limited to, red blood cells, cancer cells, stem cells and immune cells.^2^ By selecting the appropriate cell source, CNPs can be designed to interact with specific disease substrates, allowing for more precise drug delivery and reduced off‐target effects. For example, platelet membrane‐coated nanoparticles that present P‐selectin on their surface have been used to actively target breast cancer cells that express CD44.^4^ The addition of specific ligands onto the surface of CNPs can further boost their utility for drug delivery applications, and this has been accomplished using approaches such as chemical conjugation, lipid‐based anchoring, genetic engineering and metabolic engineering.^5^

In contrast to traditional nanoengineering workflows, cell membrane coating offers a relatively streamlined approach for the development of disease‐specific nanoformulations. When designing CNPs for targeted delivery applications, there is no need to undergo the time‐consuming and labour‐intensive processes of identifying and characterizing specific receptors on a cell or tissue of interest. Instead, researchers can directly take advantage of ligand–receptor interactions that have evolved over millions of years by utilizing membrane coatings that are derived from cells known to interact with a desired target. Furthermore, due to the fact that each CNP carries a diverse range of markers that can effectively interact with a variety of targets, a single formulation may be useful for multiple indications, which can also help to reduce the time and resources required for drug product development.

In preclinical cancer treatment studies, CNPs have been evaluated for their ability to augment various therapeutic modalities, including drug delivery, phototherapy and immunotherapy.^3^ Long‐circulating and targeted nanocarriers utilizing membrane coatings derived from red blood cells, platelets and cancer cells have proven to be excellent for the delivery of chemotherapeutics and other anticancer payloads. By enhancing localization to tumour sites, higher therapeutic efficacy can be achieved while concurrently reducing off‐target effects that limit the maximum drug dosage patients can tolerate. Notably, CNPs fabricated using membrane derived from cancer cells have been widely utilized for cancer drug delivery applications due to their homotypic binding properties, which enables them to target tumours derived from the same source cell.^6^ This unique approach for cancer treatment may eventually be useful for the development of personalized formulations, in which CNPs are fabricated from a patient's own resected primary tumour material in order to prevent relapse and metastasis. While CNPs have been commonly used as carriers for cytotoxic drugs, researchers have more recently been exploring the use of such platforms to deliver photothermal and photosensitizing agents.^7^ After accumulation within tumours, these nanoscale phototherapeutics can be activated by light to induce local hyperthermia or to produce reactive oxygen species, thus resulting in tumour cell killing. Compared with other systemic treatment approaches, phototherapies provide an added layer of specificity, as the therapeutic action only occurs at the irradiated tumour region. Another area in which CNPs excel is cancer immunotherapy, as they can efficiently deliver immunostimulatory agents, serve as antigen sources or directly interface with immune cells to promote antitumour immunity.^8^ Nanoparticles coated with cancer cell membranes can be used to deliver tumour material along with potent adjuvants to immune cells, thereby promoting antigen presentation and the activation of antitumour immunity.^9^ In other cases, CNPs made using immune cell membranes can directly modulate the immune system to help overcome tumour immunosuppression.^10^

While CNPs have demonstrated considerable promise for anticancer applications, certain challenges remain for clinical translation. Importantly, production must be scaled up in order to meet the needs of human patients while adhering to strict quality requirements. Various synthetic nanoparticle platforms have already been approved for human use or are in late‐stage clinical trials. For CNPs, much of the focus will likely be placed on the cell membrane derivation and coating processes, and existing industrial techniques such as microfluidization and tangential flow filtration can be adapted for cost‐effective and high‐yield production. Another consideration is the procurement of cell membrane for the preparation of CNPs. Many cell types are readily available, such as red blood cells, platelets and primary immune cells; in other cases, cell lines may be cultured in large quantities using bioreactors. All cell sources would need to be screened for transmissible diseases, and immunocompatibility with patients will be an important consideration. Finally, analytical assays need to be developed to ensure CNP quality at various stages during their manufacture. The integrity of key proteins, potency, physicochemical properties, stability and presence of microbial/viral contaminants are among some of the many parameters that will need to be evaluated.

Overall, CNPs hold immense potential as a new class of anticancer therapeutics due to their many favourable attributes, such as high biocompatibility, specific targeting ability, streamlined development and broad applicability. Development on CNPs is rapidly advancing, and considerable preclinical research has helped to validate their utility for various drug delivery, phototherapy and immunotherapy applications. While much work still needs to be done, continued efforts by researchers will undoubtedly address the challenges required for clinical translation. Ultimately, it is envisioned that CNPs may become a mainstay in the clinic, helping to significantly improve the outcomes of cancer patients.

## CONFLICT OF INTEREST STATEMENT

Liangfang Zhang holds equity interests in Cellics Therapeutics and Cello Therapeutics. The remaining authors declare no conflicts of interest.

## References

[1] Shi J , Kantoff PW , Wooster R , Farokhzad OC . Cancer nanomedicine: progress, challenges and opportunities. Nat Rev Cancer. 2017;17:20‐37. 27834398 10.1038/nrc.2016.108 PMC5575742

[2] Fang RH , Kroll AV , Gao W , Zhang L . Cell membrane coating nanotechnology. Adv Mater. 2018;30:e1706759. 29582476 10.1002/adma.201706759 PMC5984176

[3] Fang RH , Gao W , Zhang L . Targeting drugs to tumours using cell membrane‐coated nanoparticles. Nat Rev Clin Oncol. 2023;20:33‐48. 36307534 10.1038/s41571-022-00699-x

[4] Hu Q , Sun W , Qian C , Wang C , Bomba HN , Gu Z . Anticancer platelet‐mimicking nanovehicles. Adv Mater. 2015;27:7043‐7050. 26416431 10.1002/adma.201503323 PMC4998740

[5] Ai X , Wang S , Duan Y , et al. Emerging approaches to functionalizing cell membrane‐coated nanoparticles. Biochemistry. 2021;60:941‐955. 32452667 10.1021/acs.biochem.0c00343 PMC8507422

[6] Fang RH , Hu CM , Luk BT , et al. Cancer cell membrane‐coated nanoparticles for anticancer vaccination and drug delivery. Nano Lett. 2014;14:2181‐2188. 24673373 10.1021/nl500618u PMC3985711

[7] Zhen X , Cheng P , Pu K . Recent advances in cell membrane‐camouflaged nanoparticles for cancer phototherapy. Small. 2019;15:1804105. 10.1002/smll.201804105 30457701

[8] Zhou J , Kroll AV , Holay M , Fang RH , Zhang L . Biomimetic nanotechnology toward personalized vaccines. Adv Mater. 2020;32:1901255. 10.1002/adma.201901255 PMC6918015 31206841

[9] Kroll AV , Fang RH , Jiang Y , et al. Nanoparticulate delivery of cancer cell membrane elicits multiantigenic antitumor immunity. Adv Mater. 2017;29:1703969. 10.1002/adma.201703969 PMC5794340 29239517

[10] Deng G , Sun Z , Li S , et al. Cell‐membrane immunotherapy based on natural killer cell membrane coated nanoparticles for the effective inhibition of primary and abscopal tumor growth. ACS Nano. 2018;12:12096‐12108. 30444351 10.1021/acsnano.8b05292

